# Digging into the microcirculation: the rush for gold may excavate apples and oranges

**DOI:** 10.1007/s10877-016-9935-4

**Published:** 2016-10-11

**Authors:** E. Christiaan Boerma, Thomas W. L. Scheeren

**Affiliations:** 1 Department of Intensive Care, Medical Centre Leeuwarden, Henri Dunantweg 2, 8934 AD Leeuwarden, The Netherlands; 2 Department of Anesthesiology, University Medical Centre Groningen, University of Groningen, Groningen, The Netherlands

 The microcirculation is a central part of the cardiovascular system consisting of blood vessels with a diameter below 150 µm. Its main function is to exchange oxygen, carbon dioxide, nutrients, and other metabolites with the cells. The microcirculation of a healthy individual is characterized by a dense network of perfused capillaries with minimal heterogeneity, most of the capillaries being perfused even though flow in the various capillaries varies according to metabolic needs of the surrounding tissues. Adaptation to metabolic needs occurs by opening and closing capillaries, i.e. by adapting microcirculatory blood flow [1].

In recent years, a large body of knowledge pinpoints towards a pivotal pathophysiological role of the microcirculation in the development of organ failure [2]. Microcirculatory alterations are more severe in non-survivors than in survivors [3–5]. Improvements in microvascular perfusion in response to goal directed therapies are associated with subsequent improvements in organ function, while organ function further deteriorated when microvascular perfusion failed to improve or even worsened [6, 7]. Thus, the importance of monitoring the microcirculation may not be restricted to its prognostic value, but also opens up new possibilities to personalize strategies to attenuate the devastating consequences of circulatory failure in critically ill individuals.

For many years, the direct study of the microcirculation was restricted to animal experimentation. Recent introduction of video microscopy using hand-held microscopes, including orthogonal polarization spectral (OPS) imaging and side-stream dark field (SDF) imaging, allows for non-invasive visualization of the microcirculation in patients [8, 9]. A recommended off-line quantitative assessment contains a minimum set of five microvascular parameters, derived from 3 to 5 video clips, Such analysis includes parameters of vessel density (total vessel density (TVD) and perfused vessel density (PVD)), perfusion (proportion of perfused vessels (PPV) and microvascular flow index (MFI)), and heterogeneity [10]. This leads to a considerable workload, since it requires a manual analysis. Moreover, the proposed parameters fail to quantify the exact red blood cell velocity in each vessel. Both practical and theoretical aspects hold back the development of the available imaging devices from a pure off-line research tool into a real-time clinical monitoring device at the bedside, and have fuelled a ‘gold rush’ for automated software analysis.

The most commonly used software package to automatically analyse the video clips obtained with these devices is Automated Vascular Analysis^®^ (AVA). A disadvantage of AVA is the need for time consuming manual correction after automated vessel detection, in order to provide reliable parameters of vessel density. In addition, space–time frames, to quantify red blood cell velocity, can only be constructed for single vessels, with sufficient length in one direction. Thus, measurements of red blood cell velocity are selective and time consuming. Despite these limitations, Dobbe and co-workers, responsible for the development of AVA, worked along the line of solid scientific reasoning [11]. External validation with simulation videos preceded experiments with clinical images, resulting in exact numbers of accuracy and (im)precision. Finally, a comparison was made with an existing software package (CapiScope^®^) and the results presented as Bland–Altman plots.

Recently, a new device based on incident dark-field (IDF) imaging (Cytocam^®^) was developed, with improved optical lenses and a high-resolution computer controlled image sensor. This lead to enhanced detection of capillaries [12, 13]. In the wake of these developments new software was released for automatic analysis, the Cyto-Cam (CC) Tools^®^ (version 1.7.12). However, no external validation is currently (publicly) available for this new analysis software. In other words, fundamental questions, such as: what does CC Tools^®^ actually measure, and what are the accuracy and precision of these measurements as compared to a gold standard, are still to be answered.

In this issue, Carsetti and co-workers compared these two available packages of software –automated quantification of red blood cell velocity and vascular density, namely the CC Tools^®^ and AVA^®^ [14]. Under the current conditions, comparison of parameters that both software packages provide, results at best in associations, expressed as coefficients of correlation and mean bias. Carsetti et al. claim for example that this is acceptable for TVD. From a practical standpoint this may seem true. But even if the correlation is acceptable, we can still not rule out the possibility that the two software packages measure different things. For instance, what if one software package automatically includes more venules in comparison to the other, at the expense of capillaries? Then apples may look like oranges, but in fact they are not.

But it becomes more cumbersome when users of these software packages start to compare clearly different entities. The MFI is a *semi*-*quantitative* score to express the *overall* red blood cell velocity *in an area of interest* in *categories*. The drawback of such a method is obvious to the reader, yet it helps to discriminate between normal and abnormal flow by simply ‘eyeballing’ the (sublingual) microcirculation [15–17]. Comparing such a parameter with an averaged perfused speed indicator (APSI), based upon quantification of *exact* red blood cell velocity *in individual vessels, selected* by physical properties (e.g., long enough vessel) does not make sense. It at least confuses the reader and may even suggest (without saying) that one of them is false. Yet it is simply comparing apples and oranges.

We call upon the manufacturer of CC Tools^®^ to share the external validation data publicly as soon as possible. In the meantime, we urge the users of clinical in vivo microscopy not to compare apples with oranges in the rush for gold in order not to contaminate the interesting research field on the microcirculation with confusion.
